# How CGM Use in Adults With Type 2 Diabetes Leads to Glycemic Benefits: The Role of Medication and Behaviour Change

**DOI:** 10.1002/edm2.70236

**Published:** 2026-05-10

**Authors:** William H. Polonsky, Taylor L. Clark, Emily C. Soriano, Andrew Edgington, Cameron M. Bennett, Amy E. Fox, Thomas Grace

**Affiliations:** ^1^ Behavioral Diabetes Institute San Diego California USA; ^2^ Scripps Whittier Diabetes Institute, Scripps Health La Jolla California USA; ^3^ Hancock Public Health Findlay Ohio USA; ^4^ Blanchard Valley Health System Findlay Ohio USA; ^5^ Dexcom San Diego California USA

## Abstract

**Introduction:**

Although continuous glucose monitoring (CGM) in adults with type 2 diabetes (T2D) has known glycemic benefits, the underlying behavioural and psychosocial processes driving these outcomes remain poorly understood. We examined how CGM influences patient‐reported outcomes and whether changes in those outcomes predict glycemic improvement.

**Research Design and Methods:**

This 6‐month prospective observational study included 115 adults with T2D and elevated HbA1c who did not use fast‐acting insulin and initiated CGM through a real‐world program. At baseline, 3‐ and 6‐months, HbA1c, medication use/changes and key psychosocial and self‐care behaviours were assessed. Longitudinal structural equation models assessed changes over time and predictors of HbA1c reduction. Post hoc moderation analyses explored whether the effect of self‐care behaviour improvements on HbA1c outcomes depended upon new medication starts at baseline.

**Results:**

HbA1c declined significantly from 9.4% (79 mmol/mol) at baseline to 7.3% (56 mmol/mol) at both 3 and 6 months (*p*s < 0.001). Participants reported increased diabetes engagement, reduced distress, increased physical activity, fewer missed medications and less overeating (*p*s < 0.001). In multivariate models, greater HbA1c reduction was independently predicted by: (1) starting a new diabetes medication in the few months before baseline (*b* = −1.06, *p* < 0.001); (2) increases in physical activity (*b* = −0.13, *p* = 0.040); and (3) improvements in medication‐taking (*b* = 2.33, *p* < 0.001). Post hoc moderation analysis revealed that behaviour changes were most predictive of glycemic benefit among participants who had *not* started a new diabetes medication pre‐baseline.

**Conclusions:**

Real‐world CGM initiation was associated with significant improvements in glycemic control, self‐care behaviours and psychosocial outcomes. Behaviour change—notably, improved diet and physical activity—was a key contributor to glycemic gains, particularly among those not undergoing medication adjustments prior to CGM initiation. These findings support CGM as a catalyst for engagement and behaviour change in T2D management.

## Graphical abstract

This 6‐month study of CGM use in adults with T2D found greater HbA1c reduction was independently predicted by self‐care changes and by starting a new medication immediately before study start. Behaviour changes were most strongly predictive of glycemic benefit among participants who hadn't started a new medication before study start.

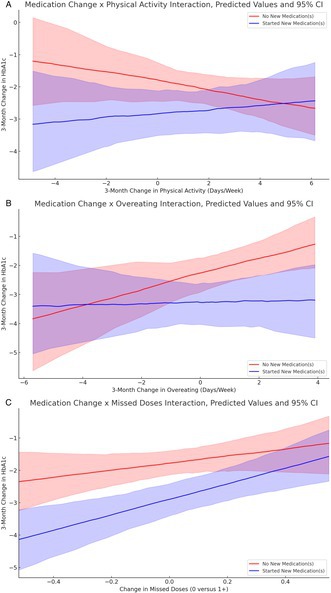

**Abbreviations**
CGM: continuous glucose monitoring
HbA1c: glycosylated haemoglobin
SMBG: self‐monitoring of blood glucose
T2D: type 2 diabetes

## 1 Introduction

Across multiple randomized controlled trials (RCTs) and observational studies, continuous glucose monitoring (CGM) use has been linked to significant glycemic benefits in poorly managed type 2 diabetes (T2D) populations [1]. As a result, the 2026 American Diabetes Association's Standards of Care now recommends early and widespread adoption of CGM for adults with T2D, even for those who are not on insulin [2]. Unfortunately, access to continuous glucose monitoring (CGM) still remains limited for people with T2D, especially those not on intensive insulin regimens, often due to high out‐of‐pocket costs, restricted or absent insurance coverage and hesitations among primary care providers.

What remains uncertain is how self‐care behaviour and/or indicators of quality of life (QOL) may be affected by CGM, and how such changes (if and when they occur) may explain the reported glycemic benefits. Results from small qualitative reports as well as larger observational studies suggest that the introduction of CGM in individuals with T2D can lead to an array of positive psychosocial changes (e.g., reductions in diabetes distress and hypoglycaemic fear) as well as healthy changes in self‐management behaviours (e.g., greater physical activity, positive changes in dietary choices, enhanced persistence with T2D medications) [3, 4, 5, 6, 7, 8, 9]; but these impacts have not yet been observed in RCTs [10, 11, 12]. Moreover, it is widely presumed that positive changes in self‐care behaviour are one of the major drivers of CGM‐induced HbA1c reductions in adults with T2D with problematic glycemic control (i.e., HbA1c above recommended targets). Once again, qualitative studies support this hypothesis [13], but we are unaware of any formal quantitative investigations in this area.

To explore these issues further and to elucidate *how* CGM use contributes to glycemic gains, we conducted a prospective observational study where adults with T2D, including insulin and non‐insulin users, were surveyed at CGM initiation, 3 and 6 months to examine changes over time in key psychosocial metrics, self‐care behaviour and glycemic outcomes. Subjects were recruited from a larger real‐world project where CGMs were provided at no cost to adults with T2D in a semi‐rural community [14]. Participants agree to attend quarterly visits where the survey batteries and HbA1c tests are completed. We hypothesized that: (1) the introduction of CGM would positively influence psychosocial and glycemic outcomes and would prompt positive changes in diabetes‐related self‐care behaviours and (2) changes in self‐care behaviour would predict glycemic outcomes.

## 2 Research Design and Methods

### 2.1 Participants

Subjects were 115 adults who were recruited from a larger real‐world project, the Dexcom Community Glucose Monitoring Project (DCGMP), where all adults with T2D with elevated glycemia (HbA1c > 7.5%/58 mmol/mol) in the regions around Hancock County, a semi‐rural area in Ohio, were eligible to receive CGM at no cost for 12 months. Participant recruitment began in February 2023 and data collection concluded in November 2024. All participants picked up CGM supplies quarterly at the local county Health Department, though this transitioned to a primary care practice in the latter months of the project. HbA1c testing was completed at each quarterly visit. Subjects were assisted by staff in setting up and starting to use their CGM systems; they were encouraged to use their systems continuously over the course of the study. Training, however, was relatively minimal; participants were provided with little guidance regarding interpretation and use of their CGM data. Six‐month results for the DCGMP focusing on glycemic outcomes were recently published [14].

For the current study, subjects from the DCMGP were determined to be eligible if they were > 21 years old, diagnosed with T2D > 6 months ago, no use of fast‐acting insulin (i.e., non‐insulin users and basal‐only insulin users were eligible) and reported no prior experience with CGM. The sample size was determined based on the number of eligible participants available during the recruitment period. Prior to receiving their initial CGM supplies at the baseline visit (nine Dexcom G7 sensors to cover the first 3 months of use), all participants were informed about the current survey study by project staff. They were told that the study was a collaboration between the Hancock County Health Department, Dexcom and the Behavioural Diabetes Institute (BDI), that survey responses would be kept anonymous, and participation was voluntary. If interested, subjects were invited to complete an online screening questionnaire at the time of the baseline visit. Individuals meeting screening criteria and providing informed consent then immediately completed the online battery of survey instruments. At the 3‐ and 6‐month visits, participants once again completed the survey battery. A $50 e‐gift card was provided after completing each survey battery ($150 in total). The research protocol for the study was independently approved by Ethical and Independent Review Services (Independence, MO), a community‐based institutional review board. The study number is 23011.

### 2.2 Measures

Key sample characteristics were captured in the first section of the survey, which included demographic information, diabetes history and relevant critical clinical metrics. These included age, gender, race/ethnicity, education, annual income, body mass index (BMI; self‐reported weight and height), number of self‐reported symptomatic hypoglycaemic episodes in the past week (i.e., where the subject noted one or more symptom, such as sweating, shakiness and/or rapid heartbeat) and current use of glucose monitoring. Also, participants reported any medication changes that had been made in the 3 months prior to CGM initiation (at baseline), and between each follow‐up time point. Specifically, items assessed whether insulin, oral diabetes medication and/or GLP‐1 receptor agonist treatment had been newly started, stopped, dose increased or decreased (or no change) in the prior 3 months.

Three items, adapted from the Summary of Diabetes Self‐Care Activities (SDSCA), were administered to assess diabetes self‐management behaviours [15]. Subjects were asked to estimate how many days over the past week (0–7) they had: missed or skipped ≥ 1 dose of their prescribed diabetes medications, exercised moderately or strenuously, and eaten more ‘than they probably should have’ (perceived overeating). This last item was developed and selected in place of the five diet‐related items from the SDSCA, as it better reflected one of the most common dietary changes reported in previous qualitative studies by T2D participants following the introduction of CGM [13, 16].

The last section of the survey assessed key psychosocial dimensions, including the 8‐item Type 2 Diabetes Distress Assessment System (T2‐DDAS) Core scale, which assesses overall diabetes distress [17], and the World Health Organization‐5 Well‐Being Index (WHO‐5), which assesses overall well‐being [18]. Together, these two measures capture essential elements of QOL among adults with T2D. In addition, four new items were developed to capture how engaged or disengaged with their diabetes care the individual might feel (‘How much attention and effort do you spend caring for your diabetes?’, ‘How concerned have you been about your diabetes?’, ‘How important has taking care of your diabetes been to you?’, ‘How confident have you felt about your ability to manage diabetes?’). Each item was scored on a ten‐point Likert scale, ranging from ‘not at all’ (1) to ‘an enormous amount’ or ‘extremely’ (10). Cronbach's alpha of the four engagement items ranged from 0.75 to 0.85 across time points, suggesting sufficient internal consistency for this new scale. Plans are now underway to document the validity of this new scale in a new study.

The baseline, 3‐month and 6‐month surveys included the same measures, except the baseline survey had additional items (e.g., demographics) that were not expected to change.

### 2.3 Data Analyses

A latent variable framework with Bayesian estimation was utilized to first model change in each clinical (HbA1c, symptomatic hypoglycaemic episodes, BMI), psychosocial (diabetes engagement, diabetes distress, overall well‐being) and self‐care behaviour (physical activity, overeating, missed diabetes medication doses) outcome over time. Two sets of growth models were estimated: one model of change from baseline to 3 months (two time points/waves) and another from baseline to 3 and 6 months (three time points/waves). Each model estimated random latent slopes, which allowed for individual differences (variance) in mean change over time. The two‐wave models estimated a latent change score representing an error‐free (latent) estimate of change in each outcome from baseline to 3 months [19]. The three‐wave model estimated latent growth curves, including a latent intercept and slope over the three time points, scaled to reflect total change over the 6‐month period. Potential covariates were examined in a preliminary model and included in all subsequent models if they emerged as predictors (*p* < 0.1) of change in HbA1c, the primary study outcome, over time. Outcomes were normally distributed except for the following: binary variables: symptomatic hypoglycaemic episodes and missed medication doses (0 vs. 1 or more); count variables: physical activity and perceived overeating (0–7 days/week). Binary outcomes were analysed using logistic regression and count outcomes with Poisson regression. Medication changes prior to CGM initiation and over the 6‐month study period were also of interest and examined using basic descriptive statistics at each time point; that is, participants reported yes/no started a new diabetes medication change in the previous 3 months (before baseline, between baseline and 3 months, and between 3 and 6 months).

After examining change in all outcomes over time, the HbA1c outcome models described above were built upon to test key predictors of HbA1c over time: First, new diabetes medication starts (insulin, oral medication and/or GLP‐1 receptor agonist) reported at baseline was examined as a focal predictor of HbA1c change over 3 and 6 months, controlling for covariates. Next, change in self‐care behaviours (physical activity, overeating, missed medication doses) was examined in the same way. Finally, to test the unique, independent effect of medication changes versus self‐care behaviour changes on HbA1c change over time, both models were combined in a multivariate model including all predictors. Post hoc two‐way interaction models examined whether starting new diabetes medication before baseline statistically moderated the association between self‐care behaviour and change in HbA1c; that is, tested two‐way interaction between baseline medication change × latent change in diabetes self‐care behaviour from baseline to 3 months. To interpret interaction effects, simple slopes were estimated and plotted separately for participants who did not start versus did start a new diabetes medication before baseline.

Analyses were conducted in Mplus 8.5 [20]. Posterior distributions were obtained using Markov Chain Monte Carlo with Gibbs sampling (10,000 iterations, thinning = 50). Non‐informative Bayesian priors were used, and 95% credibility intervals indicate statistical significance (two‐tailed *p* < 0.05) when interval did not include zero. Analyses utilized an intent‐to‐treat approach, and all available data were included regardless of missingness at follow‐up time points.

## 3 Results

### 3.1 Participant Characteristics

Of the 115 subjects who completed the baseline survey and HbA1c testing, 103 returned to complete the survey and HbA1c testing at 3 months (89.6%), and 101 did so at 6 months (87.8%). At baseline, the majority were male (57.4%), non‐Hispanic White (84.3%), 46.3% reported an annual household income < $50,000, and 19.1% were college graduates. Mean age was 57.5 (±12.9) years. All subjects had been living with T2D for ≤ 5 years. Mean BMI was 35.6 (±8.7) and mean HbA1c was 9.4% ± 1.6 (79 ± 17 mmol/mol). The majority (54.8%) were not using insulin. Symptomatic hypoglycemia was relatively infrequent, with only 28.7% reporting ≥ 1 episode in the past week. While prior CGM use was an exclusion criterion at screening, we discovered after enrollment that 16.5% of the sample (*n* = 19) had reported some type of pre‐study experience with CGM technology; however, only three individuals were regularly using CGM prior to study start (2.6% of the total sample (*n* = 115); Table 1). Note that all 19 of the CGM‐experienced participants were included in all of our analyses, as it is the least biased and most statistically conservative. However, in two separate sensitivity analyses, the main analyses were re‐run after excluding (a) the three participants with regular CGM use prior to study start and (b) the 19 participants with any prior CGM experience. In both cases, the same pattern of results emerged as those reported below.

**TABLE 1:** Baseline sociodemographic and diabetes characteristics (total sample *N* = 115).

	*N*	% of total sample
**Sociodemographic characteristics**		
Age, M (SD)	57.5	12.9
Female	49	42.6%
Race & Ethnicity		
Asian	1	0.9%
Black/African American	2	1.7%
Latino/Hispanic/Chicano	8	7.0%
Native American	1	0.9%
Non‐Hispanic White/Caucasian	97	84.3%
Multiple racial/ethnic background	2	1.7%
Other	4	3.5%
Education Level		
Less than a high school degree	5	4.3%
High school degree or equivalent (G.E.D.)	41	35.7%
Some college	47	40.9%
Bachelor's degree (e.g., B.A. or B.S.)	12	10.4%
Graduate degree or higher (e.g., Master's, MD, PhD)	10	8.7%
Income		
Under $15,000	6	5.2%
$15,000 to $24,999	5	4.3%
$25,000 to $49,999	39	33.9%
$50,000 to $74,999	27	23.5%
$75,000 to $99,999	15	13.0%
$100,000 to $149,999	12	10.4%
$150,000 and up	4	3.5%
Partnered	90	78.3%
**Baseline diabetes characteristics**		
Diagnosed with diabetes 6 months to 5 years ago	115	100.0%
Used SMBG before study	73	63.5%
Once a day	35	30.4%
Twice a day	15	13.0%
Three to four times a day	18	15.7%
Five or more times a day	5	4.3%
Any prior CGM experience	19	16.5%
Used CGM regularly in last few months	3	2.6%
Using insulin	52	45.2%
Not using insulin	63	54.8%
Oral medication only	42	36.5%
Weekly non‐insulin injectable	18	15.7%
No medication reported	3	2.6%
1+ Symptomatic hypoglycemia episodes in last week	33	28.7%
Body mass index (BMI), M (SD)	35.6	8.7

Age, gender, race/ethnicity, education, annual income and baseline insulin use were evaluated as potential covariates in two preliminary latent change models of HbA1c at 3 months and 6 months. Three variables were associated with HbA1c change (*p* ~ < 0.1) at one or both time points: Older age was related to less HbA1c decline at 3 months, *b* (SE) = 0.03 (0.01), *p* = 0.080, and 6 months, *b* = 0.03 (0.01), *p* = 0.080. Females had somewhat lower drops in HbA1c than males at 3 months, *b* = 0.55 (0.36), *p* = 0.112, and at 6 months, *b* = 0.69 (0.40), *p* = 0.080. Finally, non‐Hispanic White participants showed significantly greater declines in HbA1c than other groups at 3 months, *b* = −0.97 (0.51), *p* = 0.050, and 6 months, *b* = −0.90 (0.49), *p* = 0.020. Education, income and baseline insulin use were unrelated to HbA1c change at 3 and 6 months, all *p*s > 0.560. Therefore, in all subsequent models, age, gender, race/ethnicity were retained as covariates.

### 3.2 Changes in Clinical and Psychosocial Outcomes

Mean HbA1c fell significantly (*p* < 0.001) from baseline (9.4%) (79 mmol/mol) to 3 months (7.3%) (56 mmol/mol), with change maintained at 6 months (*p* < 0.001). The adjusted estimated total change in HbA1c was −2.1%, SE = 0.2 (−23 mmol/mol, SE = 2 mmol/mol). There were no significant changes in frequency of symptomatic hypoglycaemic episodes over the 6‐month period. A small drop in BMI occurred from baseline (M = 35.6) to 3 months (M = 35.4, *p* < 0.01), which was maintained though no longer significant at 6 months (M = 35.4, *p* = 0.096).

The three psychosocial measures (diabetes distress, diabetes engagement and overall well‐being) showed the same positive trend over time. Specifically, improvements from baseline to 3 months were observed for diabetes distress and diabetes engagement (both *p*s < 0.001), which were sustained at 6 months (both *p*s < 0.001). Gains were also observed in overall well‐being at 3 months (*p* < 0.001), though this improvement was only marginally significant at 6 months (*p* = 0.091) (Table 2).

**TABLE 2:** Clinical and psychosocial outcomes.

Outcome	Descriptives by time point			Adjusted latent change estimate			
	*N* (%) or M (SD)			Baseline–3 months		Baseline–6 months	
	Baseline	3 months	6 months	*b* (SE)	*p*	*b* (SE)	*p*
HbA1c (%) (mmol/mol)	9.4 (1.6) or 79 (18)	7.3% (1.3) or 56 (14)	7.3% (1.4) or 56 (15)	−2.13 (0.15)***	< 0.001	−2.09 (0.17)***	< 0.001
Symptomatic hypoglycemia episodes (0 vs. 1+)^a^	33 (28.7%)	28 (27.2%)	34 (33.7%)	0.69 (0.35)	0.260	1.07 (0.34)	0.840
Body mass index	35.6 (8.7)	35.4 (9.0)	35.4 (9.2)	−0.44 (0.16)**	0.004	−0.31 (0.19)	0.096
Diabetes engagement	5.5 (1.6)	6.8 (1.7)	6.8 (1.6)	1.33 (0.17)***	< 0.001	1.29 (0.16)***	< 0.001
Diabetes distress	2.6 (1.0)	2.3 (0.9)	2.2 (1.0)	−0.29 (0.09)**	0.001	−0.33 (0.09)***	< 0.001
Overall well‐being	52.0 (21.4)	58.0 (18.6)	55.6 (21.2)	5.55 (1.56)***	< 0.001	3.13 (1.86)	0.091

*Note:* Latent change estimates represent model‐implied change in the outcome, controlling for covariates (age, gender (female = 1/male = 0), race/ethnicity (non‐Hispanic White = 1/all other categories = 0)) and starting (baseline) level of the outcome. All outcome models were adjusted for age, gender and race/ethnicity.

^a^ Symptomatic hypoglycaemic episodes (experienced symptoms, in last week) analysed as categorical, binary outcomes using logistic regression; *b* replaced with exponentiated value to represent odds ratio (time scaled with one unit representing the duration examined).

** *p* < 0.01.

*** *p* < 0.001.

### 3.3 Changes in Diabetes Medications and Self‐Care Behaviours

Table 3 contains results of similar analysis of change over time in self‐care behaviours, which include moderate physical activity, perceived overeating and missed medication doses. First, there were significant increases in physical activity from baseline to 3 months, 2.1 to 2.9 days per week, *p* < 0.001, which was generally sustained at 6 months, *p* = 0.020. A similar pattern was observed for overeating behaviour, which significantly decreased from baseline to 3 months, 2.7 to 1.9 days per week, *p* < 0.001, which was sustained on average by 6 months, *p* = 0.020. Finally, the proportion of participants who endorsed ≥ 1 missed medication dose per week significantly decreased from baseline (27%) to 3 months (21%, *p* < 0.001), although this was no longer significant at 6 months (24%, *p* = 0.080).

**TABLE 3:** Behavioural outcomes.

Outcome	Descriptives by time point			Adjusted latent change estimate			
	*N* (%) or M (SD)			Baseline–3 months		Baseline–6 months	
	Baseline	3 months	6 months	*b* (SE)	*p*	*b* (SE)	*p*
Moderate physical activity, days/week^a^	2.10 (2.32)	2.93 (2.41)	2.63 (2.31)	2.16 (0.27)***	< 0.001	2.28 (0.29)*	0.020
Perceived overeating, days/week^a^	2.70 (2.26)	1.90 (1.76)	2.02 (1.48)	0.54 (0.23)***	< 0.001	0.75 (0.10)*	0.020
Missed medication doses^b^	0.27 (0.45)	0.21 (0.41)	0.24 (0.43)	0.06 (1.54)***	< 0.001	0.22 (1.047)	0.080

*Note:* Latent change estimates represent model‐implied change in the outcome, controlling for covariates (age, gender, race/ethnicity) and starting (baseline) level. Time scaled with one unit representing the duration examined.

^a^ Physical activity and perceived overeating analysed as count using Poisson regression; *b* replaced with exponentiated value to represent rate ratios.

^b^ Missed medication doses were recoded as binary, where 1 = missed 1+ medication doses in the past week, 0 = missed none; analysed as categorical, binary outcomes using logistic regression; *b* replaced with exponentiated value to represent odds ratio.

* *p* < 0.05.

*** *p* < 0.001.

Of note, many participants reported new medication starts within the 2 months prior to starting CGM at baseline: 20.9% (*n* = 24) started a new oral medication, 16.5% (*n* = 19) started a new weekly non‐insulin injectable medication, and 8.7% (*n* = 10) started basal, bolus or premixed insulin. Across the three medication categories, 39.1% (*n* = 45) started one or more new medications before baseline. However, fewer made similar changes during the 6‐month study period: At 3 months, 1.0% (*n* = 1) participant newly started insulin, 5.8% (*n* = 6) started a new oral medication, and 3.9% (*n* = 4) started a new GLP‐1 receptor agonist since baseline, with a total of 9.6% (*n* = 11) starting any new diabetes medication. Finally, the same number started new medications between 3 and 6 months: 1.0% (*n* = 1) started insulin, 5.9% (*n* = 6) started a new oral medication, and 4.0% (*n* = 4) started a new GLP‐1 receptor agonist, with a total of 8.7% (*n* = 10) starting any new diabetes medication.

### 3.4 Are Changes in Self‐Care Behaviours and/or Medications Associated With the Observed Glycemic Benefit?

Next, we examined whether changes in diabetes medications and/or self‐care behaviours predicted change in HbA1c over the study period. Changes in medication and self‐care behaviours were examined in separate models and then combined into a final multivariate model testing the independent effects of medication and self‐care behaviour change on glycemic change over time. As shown at the top of Table 4, starting any new diabetes medication in the few months before baseline significantly predicted *greater* decreases in HbA1c by nearly 1% (11 mmol/mol) over 3 months (*p* < 0.001) and 6 months (*p* = 0.020). Notably, the HbA1c drop at 6 months was estimated as −3.0% (−33 mmol/mol) for those participants who did start a new medication prior to baseline versus −1.0% (−11 mmol/mol) for those who did *not*.

**TABLE 4:** Predictors of HbA1c change over time.

Predictor	HbA1c latent change over time (Outcome)			
	Baseline–3 months		Baseline–6 months	
	*b* (SE)	*p*	*b* (SE)	*p*
**Started new medication before baseline**				
Insulin, oral medication and/or GLP‐1 receptor agonist	−0.89 (0.32)***	< 0.001	−0.89 (0.38)*	0.020
**Behaviour change over first 3 months**				
Physical activity	−0.11 (0.06)	0.060	−0.15 (0.07)**	0.006
Perceived overeating	0.12 (0.07)	0.086	0.12 (0.09)	0.180
Missed medication doses	2.02 (0.52)***	< 0.001	2.33 (0.72)***	< 0.001
**Combined multivariate model**				
Started any new medication before baseline	−0.97 (0.34)**	0.004	−1.06 (0.35)***	< 0.001
Behaviour change over first 3 months				
Physical activity	−0.13 (0.05)**	0.004	−0.13 (0.06)*	0.040
Perceived overeating	0.12 (0.07)	0.100	0.10 (0.08)	0.200
Missed medication doses	2.15 (0.54)***	< 0.001	2.33 (0.72)***	< 0.001

*Note:* Latent change estimates represent model‐implied change in the outcome, controlling for covariates (age, gender, race/ethnicity) and starting (baseline) HbA1c level. Time scaled with one unit representing the duration examined.

* *p* < 0.05.

** *p* < 0.01.

*** *p* < 0.001.

Changes in self‐care behaviour were also found, displayed in the middle of Table 4. Specifically, greater increases in physical activity over 3 months were associated with marginally greater decreases in HbA1c over the same 3‐month period (*b* = −0.11, *p* = 0.060), and significantly greater decreases in HbA1c over the full 6‐month period (*b* = −0.15, *p* = 0.006). Increases in perceived overeating over time had a similarly sized effect in the reverse direction, but were not statistically significant at 3 months (*b* = 0.12, *p* = 0.086) or 6 months (*b* = 0.12, *p* = 0.180). Changes in missed medication doses were robustly predictive of HbA1c change, such that missing fewer doses from baseline to 3 months predicted a ~2% (~22 mmol/mol) greater drop in HbA1c at 3 months (*b* = 2.02, *p* < 0.001) and 6 months (*b* = 2.33, *p* < 0.001).

The results of the combined model that included changes in self‐care behaviour *and* medications as multivariate predictors are shown in the bottom of Table 4. Overall, the same pattern of results emerged: starting a new medication prior to baseline remained a significant predictor of HbA1c change over 3 months (*p* = 0.004) and 6 months (*p* < 0.001), as were changes in physical activity (*p*s = 0.004 and 0.040) and missed medication doses (*p*s < 0.001). Each of these predictors was uniquely associated with HbA1c change, independent of the effects of the others.

### 3.5 Does Medication Change Prior to Baseline Moderate the Impact of Self‐Care Behaviour on Glycemic Outcomes?

Finally, we explored whether there is evidence of a two‐way interaction between starting a new diabetes medication before baseline and self‐care behaviour change from baseline to 3 months. We tested the interaction separately for each of the self‐care behaviours (physical activity, perceived overeating and missed medication doses). The top of Table 5 displays the results for the interaction between medication change and physical activity change, which was statistically significant in predicting 3‐month HbA1c change (*p* = 0.050), but not 6‐month HbA1c change (*p* = 0.166), despite the same unstandardized coefficient (*b* = 0.20). The middle of Table 5 shows the results for perceived overeating, which showed a similar pattern of results: a significant interaction at 3 months (*b* = −0.23, *p* = 0.040), but not at 6 months (*b* = −0.18, *p* = 0.260). The bottom of Table 5 shows the results for the interaction with missed medication doses, which was not statistically significant at 3 months (*b* = 1.35, *p* = 0.188) or 6 months (*b* = −0.03, *p* = 0.960).

**TABLE 5:** Interactions between medication and behaviour change in the prediction of HbA1c change over time.

Marginal main effect and interaction estimates (predictor)	HbA1c latent change over time (outcome)			
	Baseline–3 months		Baseline–6 months	
	*b* (SE)	*p*	*b* (SE)	*p*
**New Medication × Physical Activity Change**				
Started any new medication before baseline	−1.01 (0.35)**	0.010	−1.01 (0.41)*	0.014
Latent change in physical activity over 3 months	−0.13 (0.09)	0.110	−0.11 (0.10)	0.320
New medication × physical activity change interaction	0.20 (0.11)*	0.050	0.20 (0.14)	0.166
**New Medication × Perceived Overeating Change**				
Started any new medication before baseline	−0.97 (0.37)**	0.010	−0.93 (0.39)*	0.030
Latent change in perceived overeating over 3 months	0.26 (0.12)*	0.040	0.18 (0.13)	0.140
New medication × overeating change interaction	−0.23 (0.13)*	0.040	−0.18 (0.16)	0.260
**New Medication × Missed Doses Change**				
Started any new medication before baseline	−1.10 (0.32)***	< 0.001	−1.08 (0.40)***	< 0.001
Latent change in missed doses over 3 months	1.10 (0.79)	0.184	1.55 (1.29)	0.300
New medication × missed doses change interaction	1.35 (1.05)	0.188	−0.03 (1.77)	0.960

*Note:* Latent change estimates represent model‐implied change in the outcome, controlling for covariates (age, gender, race/ethnicity) and starting (baseline) HbA1c level. Time scaled with one unit representing the duration examined. The moderator, started any new medication before baseline, was coded as 0 = no/1 = yes in all models. No New Meds Group (*n* = 70): Participants who did not endorse starting any new diabetes medications in the 2 months before baseline; Started New Med(s) Group (*n* = 45): Participants who endorsed starting at least one new diabetes medication before baseline.

* *p* < 0.05.

** *p* < 0.01.

*** *p* < 0.001.

**FIGURE 1:**
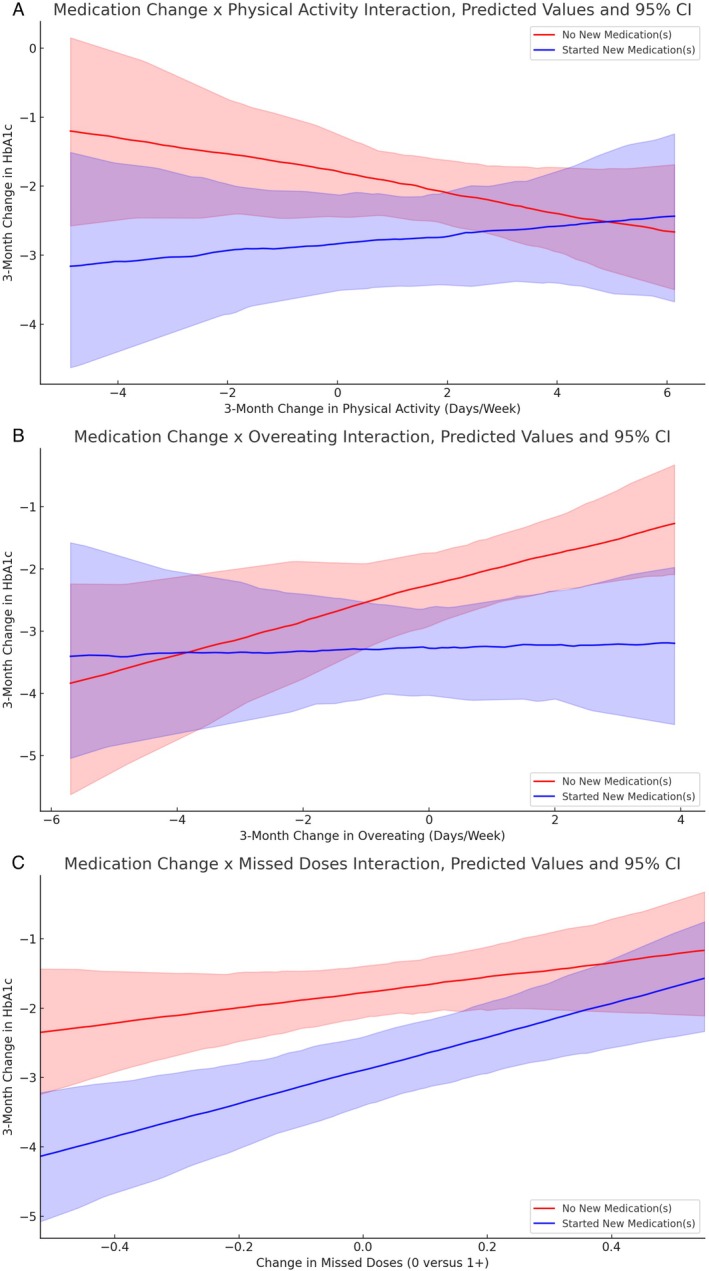
(A) Simple slope plots depicting model‐predicted association between latent change in physical activity and latent change in HbA1c over 3‐months. Among those who did not start any new medications before baseline (red line), the slope coefficient estimate (SE) = −0.13 (0.09), *p* = 0.110. Among those who did start one or more new medications before baseline (blue line), the slope estimate = 0.06 (0.11), *p* = 0.550. (B) Simple slope plots depicting model‐predicted association between latent change in perceived overeating and latent change in HbA1c over 3‐months. Among those who did not start any new medications before baseline (red line), the slope coefficient estimate (SE) = 0.26 (0.12), *p* = 0.040. Among those who did start one or more new medications before baseline (blue line), the slope estimate = 0.02 (0.14), *p* = 0.840. (C) Simple slope plots depicting model‐predicted association between latent change in missed medication doses and latent change in HbA1c over 3‐months. Among those who did not start any new medications before baseline (red line), the slope coefficient estimate (SE) = 1.10 (0.79), *p* = 0.184. Among those who did start one or more new medications before baseline (blue line), the slope estimate = 2.42 (0.66), *p* < 0.001.

As seen in the pattern of these results, illustrated in Figure 1, improvements in self‐care following CGM initiation—especially increases in physical activity and healthier eating—were associated with greater reductions in HbA1c. However, these associations were evident primarily among participants who did *not* start a new diabetes medication before baseline, suggesting that behaviour change was most predictive of glycemic improvement in the absence of concurrent medication changes.

## 4 Conclusions

Consistent with previously published results from the DCGMP [14], we found that providing CGM to adults with T2D, while providing minimal guidance and support, led to statistically significant and clinically meaningful HbA1c reductions over a 6‐month period. Though access to CGM continues to be limited for people with T2D, especially for non‐insulin users, we would hope that data from studies like these will contribute to a heightened demand for CGM use in these populations. In addition, the current findings point to significant positive changes in self‐management (fewer missed medication doses, more frequent physical activity and reductions in perceived overeating), quality of life (drops in diabetes distress and increases in overall well‐being) and subjects' sense of engagement with their own T2D care after 3 months of CGM use, most of which were also maintained after 6 months. In a qualitative study of an earlier group of DGCMP participants interviewed after 3 months of use, we similarly found that most subjects reported positive behavioural and attitudinal changes indicative of a renewed sense of interest and engagement with their own diabetes care [13]. The current prospective results support and extend these retrospective findings by documenting in a larger T2D sample over a longer period of time that these changes are significant and meaningful.

To our knowledge, this is the first report that describes *how* CGM use may be leading to glycemic improvement. In the multivariate model, we observed that three factors were independently linked with glycemic improvement at 3 months and at 6 months: having started a new diabetes medication in the 2–3 months prior to CGM initiation, increased physical activity and reductions in missed medication doses over the first 3 months of CGM use. Further analyses revealed that positive changes in eating behaviour as well as physical activity were significantly associated with glycemic benefit, but only for that group of participants (61% of the total sample) who had *not* had a new medication start shortly before beginning CGM.

It is noteworthy that few if any real‐world studies appear to have considered the potential impact of changes made immediately prior to CGM initiation on project outcomes, but such attention is clearly needed. When local clinicians selected patients with elevated HbA1c levels (> 7.5% or 58 mmol/mol) for referral to the DCGMP, we suspect that these were patients who had already been identified as requiring, and had been offered, treatment intensification. Though the baseline HbA1c values of these individuals met DCGMP's entry criteria (> 7.5% or 58 mmol/mol), the glycemic impact of these additional medications would not become apparent until the subsequent HbA1c test at 3 months. Future researchers focused on examining the impact of technology on glycemic outcomes over time, especially in real‐world settings, should be encouraged to carefully identify and track such clinician‐driven changes that may have occurred prior to study entry.

In total, these real‐world findings suggest that CGM can contribute to significant glycemic benefits in T2D populations, including insulin users as well as non‐insulin users. This is especially notable given that participants were provided with only minimal guidance and support regarding CGM use. To a considerable degree, it appears that the observed glycemic gains were due to meaningful changes in self‐management. As suggested by the earlier qualitative report of a similar DCGMP sample, these changes likely resulted not from clinician recommendations, but from participants' growing sense of engagement with their own diabetes and their own personal CGM‐induced discoveries [13]. This conclusion is supported by the significant increase in diabetes engagement observed in the current study. For clinicians seeking to enhance the benefits of CGM use among people with T2D, these findings would suggest that methods to support individuals' sense of self‐discovery, helping users to see how one's own self‐management actions and personal experiments can influence glycemia, may be particularly useful [21, 22].

Strengths of the study include its real‐world setting and the high participant retention rate over the 6‐month period. However, limitations of this study must also be considered. First, CGM was provided to all participants at no cost. Second, the majority of the sample was non‐Hispanic White, late middle age and male and all came from a relatively rural area in Ohio, which may limit the generalizability of these results to other, more diverse populations. Third, except for clinical metrics, all measures were self‐reported. While some results were based on validated instruments (diabetes distress, overall well‐being), others were adapted from other commonly‐used scales or developed specifically for this study and have yet to be validated (self‐care behaviours, engagement and medication changes). In particular, we had no access to clinic records, so reports of new medications starts (both before and during the study period) are based entirely on participant recall, which may limit their accuracy and completeness (i.e., recall bias). In addition, though prior CGM use was one of the study's exclusion criteria, our final sample inadvertently enrolled 19 participants with some prior CGM experience (including three who were using CGM regularly prior to study start), all of whom were then included in all reported analyses to minimize bias and to provide the most conservative estimates. However, it is reassuring that our two sensitivity analyses (one which excluded the 3 participants currently using CGM, and one which excluded all 19 participants with prior CGM experience) revealed the same pattern of results. Fourth, though participants were encouraged to use their CGM systems continuously over the course of the study, we do not know how well this recommendation was followed. Finally, CGM use was assessed over a relatively short time frame of 6 months, thus limiting our ability to assess long‐term maintenance of the observed glycemic and behavioural changes. As long as CGM use continues, recent evidence suggests that glycemic benefits are likely to be maintained over at least 12 months [23] but could degrade substantially if CGM is discontinued [24]. In total, all findings should be viewed with caution. Future research in this area should work to include a more diverse sample, capture all data regarding medication changes from clinic data, investigate how clinicians may be influencing participants' attitudes and actions, include more comprehensive and validated measures to assess behaviour change (e.g., wearable activity trackers), and extend the study period to ≥ 12 months.

In conclusion, we found that introducing CGM to adults with T2D was associated with significant HbA1c declines over a 6‐month period as well as significant positive changes in self‐management, quality of life and participants' engagement with their own diabetes care. Greater glycemic improvement was predicted by behaviour change as well as ≥ 1 new medication start shortly before CGM initiation. Positive changes in eating behaviour and physical activity were linked to glycemic benefit over time, but only for those who had *not* started a new medication prior to beginning CGM. These findings support and extend prior research demonstrating that CGM use in adults with T2D can positively influence glycemic outcomes and highlight that behaviour change is one critical contributor to this effect.

## Author Contributions

**Thomas Grace:** writing – review and editing. **Amy E. Fox:** investigation. **Emily C. Soriano:** writing – original draft, formal analysis, writing – review and editing. **Andrew Edgington:** investigation. **William H. Polonsky:** conceptualization, funding acquisition, writing – original draft, investigation, writing – review and editing. **Cameron M. Bennett:** investigation. **Taylor L. Clark:** investigation.

## Funding

The investigator‐initiated study was supported by Dexcom. Representatives of Dexcom had no role in the study design; in the collection, analysis and/or interpretation of the data; or in the decision to submit the manuscript for publication. An employee of the company (T.G.) was a co‐author; in this role, he was involved in the development and review of the manuscript along with the other authors. The company had no approval authority for the manuscript prior to submission, including no right to veto publication and no control on the decision regarding to which journal the manuscript was submitted.

## Conflicts of Interest

W.H.P. has served as a consultant for Dexcom and Abbott Diabetes Care. T.G. is a Dexcom employee. The other authors declare no conflicts of interest.

## Data Availability

The data that support the findings of this study are available on request from the corresponding author. The data are not publicly available due to privacy or ethical restrictions.
